# Comparison of survival outcomes with or without Para-aortic lymphadenectomy in surgical patients with stage IB1-IIA2 cervical cancer in China from 2004 to 2016

**DOI:** 10.1186/s12885-021-08797-2

**Published:** 2021-10-09

**Authors:** Chunlin Chen, Hui Duan, Wenling Zhang, Hongwei Zhao, Li Wang, Shan Kang, Lihong Lin, Weidong Zhao, Yan Ni, Donglin Li, Jiaming Chen, Huijian Fan, Xiaolin Chen, Xiaonong Bin, Jinghe Lang, Ping Liu

**Affiliations:** 1 Department of Obstetrics and Gynaecology, Nanfang Hospital, Southern Medical University, No. 1838 Guangzhou Avenue, Guangzhou, 510515 China; 2 Department of Gynaecologic Oncology, Shanxi Provincial Cancer Hospital, Taiyuan, 030013 China; 3 Department of Gynaecologic Oncology, Affiliated Tumour Hospital of Zhengzhou University, Zhengzhou, 450008 China; 4 Department of Gynaecology, Fourth Hospital Hebei Medical University, Shijiazhuang, 050019 China; 5 Department of Obstetrics and Gynaecology, The Anyang Tumor Hospital of Henan Province, Anyang, 455000 China; 6 Department of Gynaecology, Anhui Cancer Hospital, No. 17 Lujiang Avenue, HeFei, 230001 China; 7 Department of Obstetrics and Gynaecology, Yuncheng Central Hospital, Yuncheng, 044000 China; 8 Department of Obstetrics and Gynaecology, Guizhou People’s Hospital, Guiyang, 550002 China; 9 Department of Epidemiology, College of Public Health, Guangzhou Medical University, Guangzhou, 511436 China; 10 Department of Obstetrics and Gynaecology, Peking Union Medical College Hospital, Peking Union Medical College, Beijing, 100730 China

**Keywords:** Cervical cancer, Para-aortic lymphadenectomy, Metastasis, Survival outcomes, Pelvic lymph node

## Abstract

**Background:**

Current opinions on whether surgical patients with cervical cancer should undergo para-aortic lymphadenectomy at the same time are inconsistent. The present study examined differences in survival outcomes with or without para-aortic lymphadenectomy in surgical patients with stage IB1-IIA2 cervical cancer.

**Methods:**

We retrospectively compared the survival outcomes of 8802 stage IB1-IIA2 cervical cancer patients (FIGO 2009) who underwent abdominal radical hysterectomy + pelvic lymphadenectomy (*n* = 8445) or abdominal radical hysterectomy + pelvic lymphadenectomy + para-aortic lymphadenectomy (*n* = 357) from 37 hospitals in mainland China.

**Results:**

Among the 8802 patients with stage IB1-IIA2 cervical cancer, 1618 (18.38%) patients had postoperative pelvic lymph node metastases, and 37 (10.36%) patients had para-aortic lymph node metastasis. When pelvic lymph nodes had metastases, the para-aortic lymph node simultaneous metastasis rate was 30.00% (36/120). The risk of isolated para-aortic lymph node metastasis was 0.42% (1/237). There were no significant differences in the survival outcomes between the para-aortic lymph node unresected and resected groups. No differences in the survival outcomes were found before or after matching between the two groups regardless of pelvic lymph node negativity/positivity.

**Conclusion:**

Para-aortic lymphadenectomy did not improve 5-year survival outcomes in surgical patients with stage IB1-IIA2 cervical cancer. Therefore, when pelvic lymph node metastasis is negative, the risk of isolated para-aortic lymph node metastasis is very low, and para-aortic lymphadenectomy is not recommended. When pelvic lymph node metastasis is positive, para-aortic lymphadenectomy should be carefully selected because of the high risk of this procedure.

## Introduction

Cervical cancer ranks fourth for incidence and mortality in females [1]. Therefore, it is of great importance to optimize individual treatments for cervical cancer. The National Comprehensive Cancer Network (NCCN) guidelines [2] state that the main surgical procedure for stage IB1/IIA1 cervical cancer is radical hysterectomy (RH) + pelvic lymphadenectomy (PL) (category 1), with or without para-aortic lymphadenectomy (PAL) (category 2B for PAL), and the second choice is RH + PL ± PAL for stage IB2/IIA2 cervical cancer (category 2B). Using the National Cancer Database, Del Carmen et al. [3] included 3212 surgical patients with stage IA2-IB2 cervical cancer and found no statistically significant difference in the 3-year survival rates between pelvic lymph node (PLN) + para-aortic lymph node (PALN) resection and PLN resection alone (*p* = 0.69). Tsuruga et al. [4] showed that PAL did not positively impact the 5-year survival rate in 308 patients. Ayhan A et al. [5] reported the same results. Hackett TE et al. [6] suggested that surgical patients with cervical cancer stage IA2-IIA undergo PAL when PLN or PALN is suspected of metastasis.

Current opinions on whether surgical patients with stage IB1-IIA2 cervical cancer should undergo PAL at the same time are inconsistent, especially when the PLN is negative or positive. Although pelvic lymphadenectomy is generally recommended in patients with early-stage and operable cervical cancer, the role of para-aortic lymphadenectomy in these cases is less clear and has remained elusive [2, 7, 8]. In particular, all patients with lymph node metastasis of stage IB1-IIA2 cervical cancer were classified as stage IIIC in the FIGO 2018 new staging system [8], and the significance of para-aortic lymphadenectomy for patients with early cervical cancer who underwent surgery is worthy of discussion. Tsuruga et al. [4] and Finan MA et al. [9] showed that the PALN nonresection group had fewer surgical complications than the PALN resection group.

Based on the clinical diagnosis and treatment for cervical cancer in China (Four C) database, our purpose was to assess the survival outcomes with or without PAL in surgical patients with stage IB1-IIA2 cervical cancer.

## Methods

### Establishment of the China cervical cancer clinical database

This multicentre retrospective study was approved by the Ethics Committee of the Nanfang Hospital of Southern Medical University (No. NFEC-2017-135) and is registered at the International Clinical Trials Registry Platform Search Port (https://trialsearch.who.int/Trial2.aspx?TrialID=ChiCTR1800017778) under clinical trial registration number CHiCTR1800017778. This retrospective cohort study was conducted following the ethical standards adopted in the 1964 Declaration of Helsinki. The four C database was developed in collaboration with 37 hospitals in mainland China and contained 46,313 cervical cancer patients who received inpatient treatment from 2004 to 2016.

#### Data collection

Uniformly trained gynaecologists collected the data using standardized data collection and quality control procedures. Patients’ medical records and pathology and examination reports were consulted, and data, including demographics, clinicopathologic features and treatment, were collected, with specific reference to our published studies [10, 11]. Among these parameters, clinical staging was revised according to the 2009 International Federation of Gynaecology and Obstetrics (FIGO) staging standard [12]. After the data collection was completed, two gynaecologists performed independent information verification to ensure accuracy and integrated missing or incomplete data from the supplementary medical records, such as the patient’s case records and the pathology and examination reports.

#### Follow-up

Because this study was a multicentre retrospective study, trained follow-up personnel at each participating unit performed the follow-up during 1–2 telephone calls. All phone numbers were called uniformly based on the medical record management centre. The follow-up content included survival status, relapse and complications. For patients whose phone number was incorrect or if the patient could not be reached, we used the last visit or report time as the survival time and extracted tumour recurrence from outpatient medical record-related information from the hospital’s outpatient medical records, picture archiving and communication system (PACS) and clinical laboratory information system.

#### Double data input

Two specially trained gynaecologists input the same data into EpiData software and reviewed the doubtful information to ensure accuracy.

#### Data storage

After collecting all case information and follow-up data and completing double-input verification, the data were aggregated and managed by a professional to establish a unified database.

### Inclusion and exclusion criteria

The following inclusion criteria were used: (1) FIGO stage IB1-IIA2 (FIGO 2009 staging system); (2) age ≥ 18 years old; (3) biopsy or postoperative pathology confirmed as squamous cell carcinoma, adenocarcinoma or adenosquamous carcinoma; (4) no neoadjuvant chemotherapy or radiotherapy before surgery; (5) Q-M type B or type C abdominal radical hysterectomy (ARH) + PL ± PAL; and (6) complete postoperative pathological data. The following exclusion criteria were used:(1) pregnancy with cervical cancer; (2) cervical stump cancer; (3) combined with other malignancies; (4) patient was lost to follow-up; and (5) patient did not meet the inclusion criteria. The scope of PAL includes PALN biopsy, low abdominal PALN resection, and high abdominal PALN resection.

### Case-control matching

The factors included in the multivariate analysis were age, FIGO stage, whether a PALN was resected, histological type, vaginal margin, parametrial infiltration, tumour diameter, deep stromal invasion, lymphovascular invasion (LVSI), and whether the postoperative adjuvant treatment was standard. Whether the postoperative adjuvant treatment was standard was based on pathological factors according to the guidelines for treatment [2, 13]: one or more postoperative pathological high-risk factors (positive lymph nodes, parametrial infiltration or positive margins): external-beam radiation therapy + platinum-containing concurrent chemotherapy (level of evidence 1) ± vaginal brachytherapy; and intermediate-risk factors (tumour size, deep stromal invasion, LVSI) according to the “Sedlis criteria” (level of evidence 1): external-beam radiation therapy ± concurrent platinum-containing chemotherapy (simultaneous chemotherapy evidence level 2B). The definition of “inadequate” in the classification of postoperative adjuvant treatment was when there are one or more postoperative high-risk factors, postoperative adjuvant therapy would be chemotherapy only or no treatment or when there are two or more intermediate-risk factors after surgery, the postoperative adjuvant therapy would be chemotherapy only or no treatment. The definition of “over” in the classification of postoperative adjuvant treatment was when there are no postoperative high-risk factors and intermediate-risk factors or when there are no postoperative high-risk factors and only one postoperative intermediate-risk factor, the postoperative adjuvant treatment would included postoperative radiotherapy or chemoradiotherapy or chemotherapy.

Because the clinicopathological data of the PALN unresected and resected groups may have differed, we used propensity score matching (PSM)/case-control matching to balance the factors that were different between the two groups to ensure that the groups were comparable.

### Outcome evaluation

The main observation outcomes were the 5-year overall survival (OS) rate and the 5-year disease-free survival (DFS) rate between the PALN unresected and resected groups of the overall and different PLN metastasis states. OS was defined as the date of diagnosis to death from any cause or the last effective follow-up. DFS was defined as the date of diagnosis to death, relapse or the last effective follow-up.

### Statistical analysis

Data analysis was performed using SPSS statistical software (version 23.0, SPSS Inc., Chicago, IL, USA). Two independent sample *t* tests were used for continuous variables, and the *X*^*2*^ test or nonparametric test was used for categorical variables or grade variables. The log-rank test in the Kaplan-Meier (KM) method was used to compare the 5-year survival outcomes (OS, DFS) of the two groups. The Cox proportional hazards regression model was used to calculate hazard ratios (HRs) and 95% confidence intervals (CIs) for the multivariate analysis. *P* < 0.05 was considered statistically significant. Statistical experts reviewed all statistical methods and statistical processes in this study.

## Results

According to the inclusion and exclusion criteria, we ultimately selected 8802 patients with stage IB1-IIA2 cervical cancer who underwent abdominal surgery, and the median follow-up time was 41 months. Among the 8802 patients, 1618 (18.38%) patients had postoperative PLN metastasis, and 357 (4.06%) patients had PAL. The PALN metastasis rate was 10.36% (37/357). When PLN metastasized, the PALN simultaneous metastasis rate was 30.00% (36/120). The risk of isolated PALN metastasis was 0.42% (1/237).

### Overall analysis (see Table 1 and Fig. 1)

#### Comparison of survival outcomes before matching overall stage IB1-IIA2 cervical cancer patients with or without PAL

The KM analysis showed a statistically significant difference (86.8% vs 80.6%, *p* = 0.002) in the 5-year DFS rate between the PALN unresected group (*n* = 8445) and the PALN resected group (*n* = 357), perhaps because of a baseline imbalance before matching. The tumour diameter (> 4 cm), histological type (adenocarcinoma, adenosquamous carcinoma), parametrial infiltration, positive LVSI, deep stromal invasion (> 1/2) and positive PLN in the PALN resected group were all higher than those in the PALN resected group before matching. The Cox multivariate analysis showed that PALN resection was not an independent factor (HR = 1.124; 95% CI, 0.849–1.489; *p* = 0.413). The difference in the 5-year OS rates between the two groups was not statistically significant (91.5% vs. 89.2%, *p* = 0.429; HR = 0.826; 95% CI, 0.551–1.238; *p* = 0.354).

**Table 1:** Clinicopathological characteristics of overall stage IB1-IIA2 cervical cancer patients with or without para-aortic lymphadenectomy

Variables	Unmatched			Matched		
	PALN unresected (n = 8445,%)	PALN Resected (n = 357,%)	*P*-value	PALN unresected (n = 1385,%)	PALN Resected (*n* = 353,%)	*P*-value
Age (years)	48.31 ± 9.737	47.77 ± 9.436	0.310	48.48 ± 9.603	47.75 ± 9.353	0.202
FIGO stage			0.072			0.925
IB1	4779 (56.6%)	201 (56.3%)		740 (53.4%)	200 (56.7%)	
IB2	885 (10.5%)	52 (14.6%)		209 (15.1%)	50 (14.2%)	
IIA1	1898 (22.5%)	71 (19.9%)		308 (22.2%)	71 (20.1%)	
IIA2	613 (7.3%)	28 (7.8%)		105 (7.6%)	27 (7.6%)	
IB	123 (1.5%)	2 (0.6%)		9 (0.6%)	2 (0.6%)	
IIA	147 (1.7%)	3 (0.8%)		14 (1.0%)	3 (0.8%)	
Tumour size			0.018			0.886
≤ 4 cm	6677 (79.1%)	272 (76.2%)		1048 (75.7%)	271 (76.8%)	
> 4 cm	1498 (17.7%)	80 (22.4%)		314 (22.7%)	77 (21.8%)	
Unknown	270 (3.2%)	5 (1.4%)		23 (1.7%)	5 (1.4%)	
Histological type			< 0.001			0.498
SCC	7587 (89.8%)	289 (81.0%)		1154 (83.3%)	287 (81.3%)	
AC	656 (7.8%)	52 (14.6%)		168 (12.1%)	51 (14.4%)	
SAC	202 (2.4%)	16 (4.5%)		63 (4.5%)	15 (4.2%)	
Parametrial			< 0.001			0.634
Negative	8307 (98.4%)	338 (94.7%)		1330 (96.0%)	337 (95.5%)	
Positive	138 (1.6%)	19 (5.3%)		55 (4.0%)	16 (4.5%)	
Vaginal margin			0.009			0.375
Negative	8250 (97.7%)	341 (95.5%)		1343 (97.0%)	339 (96.0%)	
Positive	195 (2.3%)	16 (4.5%)		42 (3.0%)	14 (4.0%)	
LVSI			< 0.001			0.979
Negative	6887 (81.6%)	262 (73.4%)		1025 (74.0%)	261 (73.9%)	
Positive	1558 (18.4%)	95 (26.6%)		360 (26.0%)	92 (26.1%)	
Cervical invasion			0.001			0.898
≤ 1/2	3289 (38.9%)	108 (30.3%)		415 (30.0%)	108 (30.6%)	
> 1/2	4598 (54.4%)	230 (64.4%)		902 (65.1%)	226 (64.0%)	
Unknown	558 (6.6%)	19 (5.3%)		68 (4.9%)	19 (5.4%)	
POAT			0.375			0.903
Standard	4619 (54.7%)	207 (58.0%)		811 (58.6%)	204 (57.8%)	
Inadequate	736 (8.7%)	35 (9.8%)		145 (10.5%)	34 (9.6%)	
Over	2927 (34.7%)	110 (30.8%)		407 (29.4%)	110 (31.2%)	
Unknown	163 (1.9%)	5 (1.4%)		22 (1.6%)	5 (1.4%)	
PLN metastasis			< 0.001			0.462
Negative	6947 (82.3%)	237 (66.4%)		901 (65.1%)	237 (67.1%)	
Positive	1498 (17.7%)	120 (33.6%)		484 (34.9%)	116 (32.9%)	

*PALN* para-aortic lymph node, *PLN* pelvic lymph node, *FIGO* International Federation of Gynaecology and Obstetrics, *SCC* squamous cell carcinoma, *AC* adenocarcinoma, *SAC* adenosquamous carcinoma, *LVSI* lymphatic vessel space, *POAT* postoperative adjuvant treatment

**Fig. 1:**
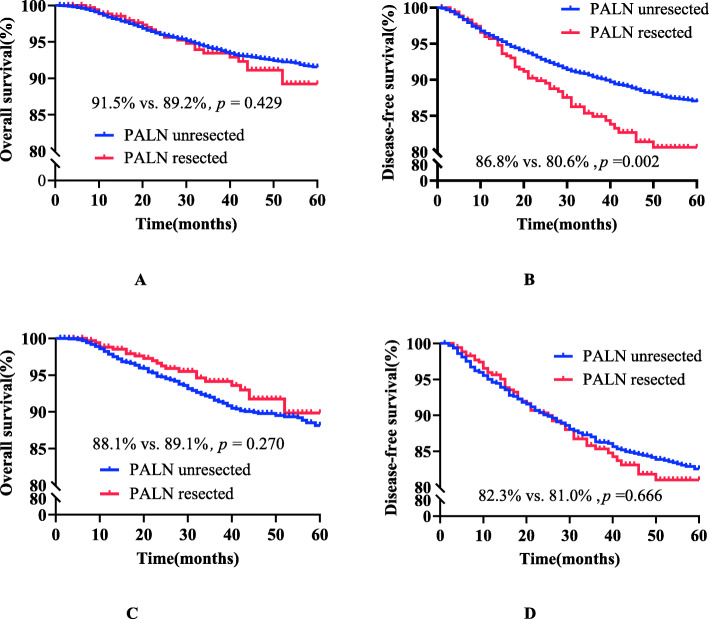
Survival curves of overall stage IB1 and IIA2 cervical cancer patients before and after matching *Before matching, panels A and B; after matching,panels C and D; *PALN* para-aortic lymph node

#### Comparison of survival outcomes after matching overall stage IB1-IIA2 cervical cancer patients with or without PAL

After 1:4 PSM matching, there was no significant difference in 5-year survival outcomes between the PALN unresected group (*n* = 1385) and the PALN resected group (*n* = 353) (OS 88.1% vs. 89.9%, *p* = 0.270; HR = 0.807; 95% CI, 0.517–1.259; *p* = 0.344; DFS 82.3% vs. 81.0%, *p* = 0.666; HR = 1.096; 95% CI, 0.805–1.491; *p* = 0.562).

### Different PLN metastasis states

#### Comparison of PALN survival outcomes before and after matching in patients with negative PLN metastasis of stage IB1-IIA2 cervical cancer with or without PAL (see Table 2 and Fig. 2)

In total, 7184 patients with stage IB1-IIA2 cervical cancer and negative PLN metastasis met the screening criteria. When PLN metastasis was negative, there was no significant difference in 5-year survival outcomes between the PALN unresected group (*n* = 6947) and the PALN resected group (*n* = 237) (OS 94.5% vs. 96.2%, *p* = 0.291; HR = 0.585; 95% CI, 0.260–1.316; *p* = 0.195; DFS 90.6% vs. 88.7%, *p* = 0.371; HR = 1.130; 95% CI, 0.772–1.769; *p* = 0.593). After 1:4 PSM matching, there was no statistically significant difference in 5-year survival outcomes between the PALN unresected group (*n* = 948) and the resected group (n = 237) (OS 94.5% vs. 96.2%, *p* = 0.292; HR = 0.599; 95% CI, 0.254–1.414; *p* = 0.242; DFS 90.7% vs. 88.7%, *p* = 0.474; HR = 1.139; 95% CI, 0.693–1.873; *p* = 0.607).

**Table 2:** Clinicopathological characteristics of negative PLN metastasis in stage IB1-IIA2 cervical cancer patients with or without para-aortic lymphadenectomy

Variables	Unmatched			Matched		
	PALN unresected (n = 6947,%)	PALN Resected (n = 237,%)	*P*-value	PALN unresected (n = 948,%)	PALN Resected (n = 237,%)	*P*-value
Age (years)	48.30 ± 9.773	47.27 ± 9.262	0.108	47.85 ± 9.597	47.27 ± 9.262	0.404
FIGO stage			0.59			0.749
IB1	4192 (60.3%)	148 (62.4%)		583 (61.5%)	148 (62.4%)	
IB2	652 (9.4%)	27 (11.4%)		129 (13.6%)	27 (11.4%)	
IIA1	1491 (21.5%)	48 (20.3%)		176 (18.6%)	48 (20.3%)	
IIA2	399 (5.7%)	9 (3.8%)		48 (5.1%)	9 (3.8%)	
IB	111 (1.6%)	2 (0.8%)		4 (0.4%)	2 (0.8%)	
IIA	103 (1.5%)	3 (1.3%)		8 (0.8%)	3 (1.3%)	
Tumour size			0.7			0.304
≤ 4 cm	5683 (81.8%)	196 (82.7%)		759 (80.1%)	196 (82.7%)	
> 4 cm	1051 (15.1%)	36 (15.2%)		177 (18.7%)	36 (15.2%)	
Unknown	213 (3.1%)	5 (2.1%)		12 (1.3%)	5 (2.1%)	
Histological type			< 0.001			0.798
SCC	6255 (90.0%)	189 (79.7%)		761 (80.3%)	189 (79.7%)	
AC	539 (7.8%)	39 (16.5%)		159 (16.8%)	39 (16.5%)	
SAC	153 (2.2%)	9 (3.8%)		28 (3.0%)	9 (3.8%)	
Parametrial			0.051			0.059
Negative	6886 (99.1%)	232 (97.9%)		941 (99.3%)	232 (97.9%)	
Positive	61 (0.9%)	5 (2.1%)		7 (0.7%)	5 (2.1%)	
Vaginal margin			0.142			0.668
Negative	6808 (98.0%)	229 (96.6%)		921 (97.2%)	229 (96.6%)	
Positive	139 (2.0%)	8 (3.4%)		27 (2.8%)	8 (3.4%)	
LVSI			0.603			0.836
Negative	6003 (86.4%)	202 (85.2%)		813 (85.8%)	202 (85.2%)	
Positive	944 (13.6%)	35 (14.8%)		135 (14.2%)	35 (14.8%)	
Cervical invasion			0.601			0.525
≤ 1/2	3067 (44.1%)	99 (41.8%)		431 (45.5%)	99 (41.8%)	
> 1/2	3381 (48.7%)	123 (51.9%)		453 (47.8%)	123 (51.9%)	
Unknown	499 (7.2%)	15 (6.3%)		64 (6.8%)	15 (6.3%)	
POAT			0.354			0.914
Standard	3486 (50.2%)	106 (44.7%)		422 (44.5%)	106 (44.7%)	
Inadequate	371 (5.3%)	16 (6.8%)		56 (5.9%)	16 (6.8%)	
Over	2927 (42.1%)	110 (46.4%)		444 (46.8%)	110 (46.4%)	
Unknown	163 (2.3%)	5 (2.1%)		26 (2.7%)	5 (2.1%)	

*PALN* para-aortic lymph node, *PLN* pelvic lymph node, *FIGO* International Federation of Gynaecology and Obstetrics, *SCC* squamous cell carcinoma, *AC* adenocarcinoma, *SAC* adenosquamous carcinoma, *LVSI* lymphatic vessel space, *POAT* postoperative adjuvant treatment

**Fig. 2:**
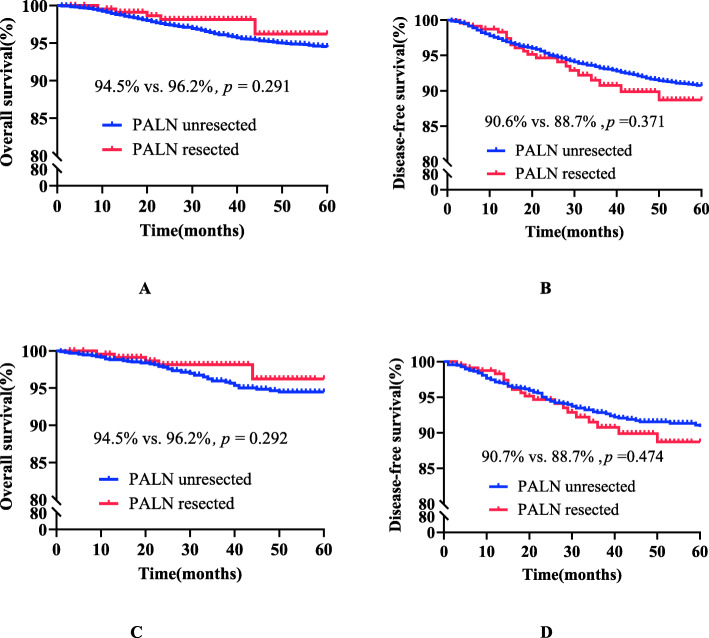
Survival curves of negative PLN metastasis in stage IB1 and IIA2 cervical cancer patients before and after matching *Negative PLN: before matching, panels A and B; after matching,panels C and D; *PALN* para-aortic lymph node; *PLN* pelvic lymph node

#### Comparison of PALN survival outcomes before and after matching in patients with positive PLN metastasis of stage IB1-IIA2 cervical cancer with or without PAL (see Table 3 and Fig. 3)

In total, 1618 patients with stage IB1-IIA2 cervical cancer and positive PLN metastasis met the screening criteria. There was no statistically significant difference in 5-year survival outcomes between the PALN unresected group (*n* = 1498) and the PALN resected group (*n* = 120) (OS 77.6% vs. 75.9%, *p* = 0.953; HR = 0.955; 95% CI, 0.596–1.532; *p* = 0.849; DFS 69.0% vs. 65.2%, *p* = 0.367; HR = 1.104; 95% CI, 0.769–1.585; *p* = 0.593). After 1:4 PSM matching, there was no statistically significant difference in 5-year survival outcomes between the PALN unresected group (*n* = 471) and the resected group (n = 120) (OS 74.2% vs. 75.9%, *p* = 0.594; HR = 0.811; 95% CI, 0.491–1.340; *p* = 0.414; DFS 67.6% vs. 65.2%, *p* = 0.733; HR = 0.996; 95% CI, 0.676–1.469; *p* = 0.985).

**Table 3:** Clinicopathological characteristics of positive PLN metastasis in stage IB1-IIA2 cervical cancer patients with or without para-aortic lymphadenectomy

Variables	Unmatched			Matched		
	PALN unresected (n = 1498,%)	PALN Resected (n = 120,%)	*P*-value	PALN unresected (n = 471,%)	PALN Resected (n = 120,%)	*P*-value
Age (years)	48.31 ± 9.573	48.77 ± 9.732	0.617	47.20 ± 9.649	47.75 ± 9.609	0.666
FIGO stage			0.073			0.814
IB1	587 (39.2%)	53 (44.2%)		215 (45.6%)	53 (44.2%)	
IB2	233 (15.6%)	25 (20.8%)		84 (17.8%)	25 (20.8%)	
IIA1	407 (27.2%)	23 (19.2%)		85 (18.0%)	23 (19.2%)	
IIA2	214 (14.3%)	19 (15.8%)		87 (18.5%)	19 (15.8%)	
IB	12 (0.8%)	0 (0.0%)		–	–	
IIA	45 (3.0%)	0 (0.0%)		–	–	
Tumour size			0.04			0.942
≤ 4 cm	994 (66.4%)	76 (63.3%)		300 (63.7%)	76 (63.3%)	
> 4 cm	447 (29.8%)	44 (36.7%)		11 (36.3%)	44 (36.7%)	
Unknown	57 (3.8%)	0 (0.0%)		–	–	
Histological type			0.152			0.077
SCC	1332 (88.9%)	100 (83.3%)		426 (90.4%)	100 (83.3%)	
AC	117 (7.8%)	13 (10.8%)		27 (5.7%)	13 (10.8%)	
SAC	49 (3.3%)	7 (5.8%)		18 (3.8%)	7 (5.8%)	
Parametrial			0.003			0.846
Negative	1421 (94.9%)	106 (88.3%)		419 (89.0%)	106 (88.3%)	
Positive	77 (5.1%)	14 (11.7%)		52 (11.0%)	14 (11.7%)	
Vaginal margin			0.113			0.265
Negative	1442 (96.3%)	112 (93.3%)		451 (95.8%)	112 (93.3%)	
Positive	56 (3.7%)	8 (6.7%)		20 (4.2%)	8 (6.7%)	
LVSI			0.054			0.852
Negative	882 (59.0%)	60 (50.0%)		240 (51.0%)	60 (50.0%)	
Positive	614 (41.0%)	60 (50.0%)		231 (49.0%)	60 (50.0%)	
Cervical invasion			0.077			0.085
≤ 1/2	222 (14.8%)	9 (7.5%)		72 (15.3%)	9 (7.5%)	
> 1/2	1217 (81.2%)	107 (89.2%)		386 (82.0%)	107 (89.2%)	
Unknown	59 (3.9%)	4 (3.3%)		13 (2.8%)	4 (3.3%)	
POAT			0.035			0.57
Standard	1133 (75.6%)	101 (84.2%)		386 (82.0%)	101 (84.2%)	
Inadequate	365 (24.4%)	19 (15.8%)		85 (18.0%)	19 (15.8%)	

*PALN* para-aortic lymph node, *PLN* pelvic lymph node, *FIGO* International Federation of Gynaecology and Obstetrics, *SCC* squamous cell carcinoma, *AC* adenocarcinoma, *SAC* adenosquamous carcinoma, *LVSI* lymphatic vessel space, *POAT* postoperative adjuvant treatment

**Fig. 3:**
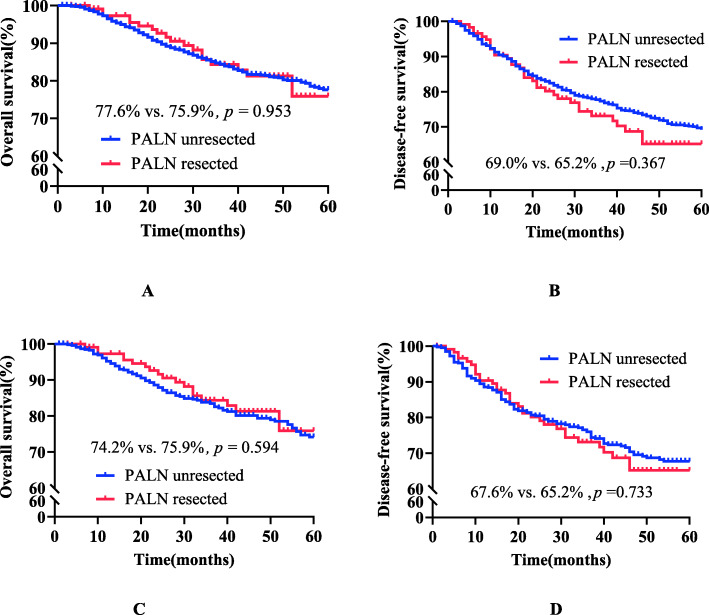
Survival curves of positive PLN metastasis in stage IB1 and IIA2 cervical cancer patients before and after matching *Positive PLN: before matching, panels A and B; after matching,panels C and D; *PALN* para-aortic lymph node; *PLN* pelvic lymph node

## Discussion

This study included 8802 patients with cervical cancer stage IB1-IIA2 who underwent ARH + PL, and 357 patients (4.06%) who underwent PAL. In patients who underwent PAL, the rate of PALN isolated metastasis was 0.42% (1/237) when the PLN was negative. In contrast, when the PLN was positive, the rate of concurrent PALN metastasis was 30.00% (36/120). Our findings highlight the low risk of isolated PALN metastasis in patients with early operable cervical cancer. Notably, there was no statistically significant difference in 5-year survival outcomes between the ARH + PL and ARH + PL + PAL groups of patients with stage IB1-IIA2 cervical cancer.

This result is consistent with a retrospective study by Del Carmen et al. [3], who reported no statistically significant difference in 3-year survival outcomes between patients receiving PLN + PALN resection and patients receiving PLN resection alone (*p* = 0.69). Ayhan A et al. [5] also suggested no statistically significant difference in 5-year survival outcomes between patients with early cervical cancer who underwent PAL and patients who did not undergo PAL.

Tsuruga et al. [4] retrospectively analysed 308 patients undergoing surgery for stage IB2, IIA2 or IIB cervical cancer. PAL failed to improve the oncological outcome. Among 30 patients with total iliac lymph node metastasis, the OS rate in the PAL group was relatively high (*p* = 0.053), but the number of patients studied was small. Hackett TE et al. [6] reported that all patients with stage IA2-IIA cervical cancer should undergo RH + PL without PAL, except for suspected metastasis of PLN or PALN. Our study found that when PLN metastasis was negative or positive, patients with stage IB1-IIA2 cervical cancer who received PAL did not show improved 5-year survival outcomes. However, patients with positive PALNs received adjuvant radiotherapy and chemotherapy, and it was not possible to evaluate whether PAL conferred a survival benefit. Lymph nodes are an important factor in the prognosis of patients, and the new FIGO staging in 2018 [8] classified patients with lymph node metastasis into stage IIIC. Whether PAL should be performed during the surgical treatment of stage IB1-IIA2 cervical cancer under the new staging classification should be considered.

The rate of isolated PALN metastasis was low, which supports the recommendation that PLN-negative patients should not undergo this procedure because the associated complications may increase when lymph node resection is extended. Recent data suggested that sentinel lymph node biopsy may be useful for decreasing the need for PL in patients with early-stage cervical cancer [2]. Tsuruga et al. [4] suggested that long-term complications in the PALN unresected group (*n* = 119) were lower than those in the PALN resected group (*n* = 135) in early cervical cancer patients who underwent RH + PL. The incidences of lymphedema, lymphocysts, and small intestinal or colonic obstruction in the two groups were 6.7%/14.1, 1.7%/4.4%, and 16/15%, respectively. Finan MA et al. [9] noted that PALN resection was the only independent predictor of surgical complications of early cervical cancer.

The present study analysed the survival outcomes of PALN resection during abdominal surgery to eliminate the interference caused by different surgical approaches [14]. Notably, Liang et al. [15] retrospectively found that laparoscopic surgery had more surgical complications than abdominal surgery. We found that the PALN resection rate of stage IB1-IIA2 cervical cancer patients was 27.27% (1104/4048) following laparoscopic surgery in this database, which was significantly higher than the rate following abdominal surgery (4.06%, 357/8802). Therefore, abdominal surgery for cervical cancer could reduce PAL and complications. The omission of PAL in patients with pathologically negative PLNs may be of great significance to the reduction of surgery-related complications.

The advantages of this study are as follows. First, our study is one of the first population-based studies to compare 5-year OS and DFS after treatment with or without para-aortic lymphadenectomy in surgical patients with stage IB1-IIA2 cervical cancer and related subgroups. Second, the strength of the present study was its large sample size. Our study analysed a large cohort of cervical cancer patients who were treated over a 12-year period at 37 hospitals. Finally, patients who underwent laparoscopic surgery were excluded from our study because the 2018 LACC study [14] indicated that laparoscopic surgery is not conducive to the survival outcomes of patients with cervical cancer.

The present study has the following limitations. First, it was a retrospective study, and there may be selection bias. Why some patients did or did not undergo PAL cannot be determined. Second, due to space limitations, the comparison of complications between the PALN unresected group and the resected group of stage IB1-IIA2 cervical cancer patients was not included in this paper. In addition, this study focused on whether patients with ARH + PL treatment in stage IB1-IIA2 cervical cancer should undergo PAL, and could not be extrapolated to whether patients with staging procedures should undergo PAL. Finally, PAL was defined as PALN biopsy and low and high PALN dissection. Different PALN resection ranges may affect patient survival outcomes.

## Conclusions

In summary, the results of this study suggest that the risk of PALN isolated metastasis is very low when the PLN is negative, and PAL is not recommended in surgical patients with stage IB1-IIA2 cervical cancer. When the PLN was positive, PAL did not significantly improve the prognosis, and PAL was associated with more complications and higher risks. Therefore, PAL should be selected carefully. This issue requires deeper prospective studies to verify.

## Data Availability

The datasets used and/or analysed for the current study are available from the corresponding author upon reasonable request.

## Abbreviations

NCCN: National Comprehensive Cancer Network
RH: radical hysterectomy
PL: pelvic lymphadenectomy
PAL: para-aortic lymphadenectomy
PLN: pelvic lymph node
PALN: para-aortic lymph node
FIGO: International Federation of Gynaecology and Obstetrics
LVSI: lymphovascular invasion
PSM: propensity score matching
